# Dietary glycemic index and glycemic load in relation to HbA1c in Japanese obese adults: a cross-sectional analysis of the Saku Control Obesity Program

**DOI:** 10.1186/1743-7075-9-79

**Published:** 2012-09-10

**Authors:** Maki Goto, Akemi Morita, Atsushi Goto, Satoshi Sasaki, Naomi Aiba, Takuro Shimbo, Yasuo Terauchi, Motohiko Miyachi, Mitsuhiko Noda, Shaw Watanabe

**Affiliations:** 1 Department of Diabetes Research, Diabetes Research Center, National Center for Global Health and Medicine, Tokyo, Japan; 2 Department of Endocrinology and Metabolism, Yokohama City University Graduate School of Medicine, Yokohama, Japan; 3 National Institute of Health and Nutrition, Tokyo, Japan; 4 Department of Nutrition, College of Nutrition, Koshien University, Hyogo, Japan; 5 Department of Social and Preventive Epidemiology, School of Public Health, University of Tokyo, Tokyo, Japan; 6 Department of Nutrition and Life Science, Kanagawa Institute of Technology, Kanagawa, Japan; 7 Department of Clinical Research and Informatics, National Center for Global Health and Medicine, Tokyo, Japan; 8 Department of Diabetes and Metabolic Medicine, Center Hospital, National Center for Global Health and Medicine, Tokyo, Japan

**Keywords:** Glycemic index, Glycemic load, HbA1c, Fasting plasma glucose, Metabolic risk factors, Obesity, Japanese

## Abstract

**Background:**

Dietary glycemic index or load is thought to play an important role in glucose metabolism. However, few studies have investigated the relation between glycemic index (GI) or load (GL) and glycemia in Asian populations. In this cross-sectional analysis of a randomized controlled trial, the Saku Control Obesity Program, we examined the relation between the baseline GI or GL and glycemia (HbA1c and fasting plasma glucose [FPG] levels), insulin resistance (HOMA-IR), β-cell function (HOMA-β), and other metabolic risk factors (lipid levels, diastolic and systolic blood pressure, and adiposity measures).

**Methods:**

The participants were 227 obese Japanese women and men. We used multiple linear regression models and logistic regression models to adjust for potential confounding factors such as age, sex, visceral fat area, total energy intake, and physical activity levels.

**Results:**

After adjustments for potential confounding factors, GI was not associated with HbA1c, but GL was positively associated with HbA1c. For increasing quartiles of GI, the adjusted mean HbA1c were 6.3%, 6.7%, 6.4%, and 6.4% (*P* for trend = 0.991). For increasing quartiles of GL, the adjusted mean HbA1c were 6.2%, 6.2%, 6.6%, and 6.5% (*P* for trend = 0.044). In addition, among participants with HbA1c ≥ 7.0%, 20 out of 28 (71%) had a high GL (≥ median); the adjusted odds ratio for HbA1c ≥ 7.0% among participants with higher GL was 3.1 (95% confidence interval [CI] = 1.2 to 8.1) compared to the participants with a lower GL (<median). Further, among 16 participants with FPG ≥ 150 mg/dL, 13 participants (81.3%) had a higher GL; the adjusted odds ratio for FPG ≥ 150 mg/dL among participants with a higher GL was 8.5 (95% confidence interval = 1.7 to 43.4) compared to those with a lower GL. In contrast, GI and GL were not associated with metabolic risk factors other than glycemia.

**Conclusions:**

Our findings suggest that participants with poor glycemic control tend to have a higher GL in an obese Japanese population.

## Background

The prevalence of abnormal glucose tolerance is increasing, and is a growing public health concern [1]. Although uncertainty exists regarding the intake of carbohydrates and glycemia, the utility of glycemic index (GI) and glycemic load (GL) has been proposed. GI measures the ability of a carbohydrate-containing food to raise the blood glucose level [2], and GL is the product of GI and the amount of carbohydrate in the food [3]. Though a lower GI or GL has been hypothesized to decrease the risk of type 2 diabetes, evidence regarding the role of GI and GL in relation to the risk of diabetes remains inconclusive. Several studies have reported that higher GI and GL values were associated with an increased risk of diabetes [4-7], but other studies have not confirmed these results [8-10].

Furthermore, because the main sources of GL differ across ethnicities, the associations between GI, GL, and diabetes may differ in Asian populations, in which rice is the major staple food [11]. The high intake of rice, has been reportedly associated with an increased risk of type 2 diabetes mellitus in Asian population [5,12,13], and also, in a study of Japanese female farmers, GI and GL independently correlated with glucose, HbA1c, body mass index (BMI), and fasting triglyceride (TG) [14]. Studies among Japanese Brazilians have also associated higher intakes of fruit, fruit juice, white bread, and rice with glucose intolerance [15,16]. However, comprehensive investigations of the relations of GI and GL with metabolic risk factors in Asian are few, and most of the preceding studies in Asian populations have been limited to women. For these reasons, in the present study, we aimed to investigate whether low dietary GI and GL values are associated with reduced glucose measures (HbA1c and FPG levels) and other metabolic risk factors, including adiposity measures (BMI, waist circumference, visceral fat area, and subcutaneous fat area), lipid levels (LDL, HDL, and TG), and blood pressures (systolic blood pressure [SBP] and diastolic blood pressure [DBP]), in a Japanese population.

## Methods

### Study population

This study is a cross-sectional analysis of a randomized controlled trial, the Saku Control Obesity Program (SCOP), examining the effect of behavioral treatment and exercise at the Saku Central Hospital Human Dock Center. The details and design of the study have been previously described elsewhere [17-19]. Briefly, the program consisted of a randomized intervention trial using cognitive-behavioral treatment at the Saku Health Dock Center. Among 976 members who visited health checkups, members with a BMI in the upper five percentile and without history of type 1 diabetes, stroke, cardiovascular disease, advanced cancer, or significant renal or hepatic dysfunction were invited. In total, 237 women and men participated. We used the baseline data for the analysis. Of the 237 people participated in the study, 10 participants did not complete the study, and 227 participants were included in the analysis. For the multiple linear regression analysis, we further excluded one participant with missing data. Also, for the analysis of the FPG and lipid levels, we excluded 3 participants who did not provide fasting blood samples. The research plan was reviewed and approved by the Ethical Committee of the National Institute of Health and Nutrition and Saku Central Hospital. Participants received a precise explanation of the study and provided their written informed consent.

### Anthropometric measurements

The height (cm) and weight (kg) of the subjects were measured with an automatic scale (Tanita, BF-220, Tokyo, Japan), in light clothing. The BMI was calculated as the weight (kg) divided by the squared height (m^2^). Waist circumference was measured twice at the umbilicus level while the subject was in a standing position using a fiber glass measuring tape; the average measurement was used for the analysis. Blood pressure was measured while the subject was in a sitting position using a validated automated blood pressure monitor (HEM-907; Omron, Kyoto, Japan) [20].

Visceral fat and subcutaneous fat areas were assessed using a computed tomography scan at the level of the umbilicus in a supine position (Fat scan; N2 system Corp., Japan). The coefficients of variation (CV) between two observers for the visceral fat area and subcutaneous fat area measurements were reported to range from 0.6% to 14.2% and from 0.1% to 7.3%, respectively [21,22]. The physical activity levels were obtained by asking the participants about their average physical activity levels for the past month. The physical activity levels were divided into four levels: light activity (sedentary labor most of the day, including 1 hour walking, or standing for approximately 3 hours), light to moderate activity (between sedentary and manual labor, including walking for about 2 hours or standing for 6 to 7 hours), moderate activity (manual labor for approximately 1 hour, including walking or standing for approximately 9 hours, with hard activity for 1 hour), and vigorous activity (manual labor, walking or standing for approximately 9 hours, with hard activity for more than 2 hours).

### Laboratory procedures

Following an overnight fast, blood samples were collected at the time of each health checkup at the Saku Health Dock Center. Blood samples were collected in tubes containing EDTA and heparin for the measurement of the fasting plasma glucose, insulin, and HbA1c levels, and serum gel separator tubes were used for the measurement of the total cholesterol, HDL cholesterol, and TG levels. Routine laboratory blood analyses were performed at the Saku Central Hospital. HbA1c levels were measured using a high-performance liquid chromatography method (TOSOH HLC-723 G8; Tosoh Corporation, Tokyo, Japan), with intra- and inter-assay coefficients of variation (CVs) of 0.5%-1.4% and 0.6%-1.3%, respectively. The plasma glucose levels were analyzed using an enzymatic method (ECO glucose buffer; A&T Corporation, Kanagawa, Japan), with intra- and inter-assay CVs of 0.3%-0.5% and 0.6%-0.8%, respectively. The plasma insulin levels were analyzed using an electrochemiluminescence immunoassay (Modular E170; Roche Diagnostics, Mannheim, Germany), with intra- and inter-assay CVs of 0.5%-2.0% and 3.2%-3.6%, respectively. The serum total cholesterol, HDL cholesterol, and TG concentrations were determined using enzymatic methods (serum total cholesterol: Detaminar L TC II, Kyowa Medex, Tokyo, Japan; HDL cholesterol: Cholestest N HDL,Sekisui Medical Co. Ltd., Tokyo, Japan; and TG concentrations: Mizuho TG-FR Type II, Mizuho Medi, Saga, Japan) and an autoanalyzer BM-2250 (Nihon Denshi, Tokyo, Japan), with intra- and inter-assay CVs of ≤1.7% and ≤2.3%, respectively.

The value for HbA1c (%) was estimated as an NGSP equivalent value (%) calculated by the formula HbA1c (%) = HbA1c (JDS) (%) + 0.4%, considering the relational expression of HbA1c (JDS) (%) measured by the previous Japanese standard substance and measurement methods and HbA1c (NGSP) [23]. The homeostasis model assessment for insulin resistance (HOMA-IR) and homeostasis model assessment for β cell function (HOMA-β) were calculated as follows: HOMA-IR = fasting insulin (μIU/ml) × fasting glucose (mmol/ml) / 22.5, and HOMA-β = 20 × fasting insulin (μIU/ml) / [fasting glucose (mmol/ml) – 3.5] [24,25].

### Assessment of dietary intake

Dietary habits were assessed using a previously validated, self-administered diet history questionnaire (DHQ). The methods used to calculate dietary intake and the validity of the DHQ have been published elsewhere [26-29]. In brief, the DHQ consists of a 16-page questionnaire for assessing dietary habits during the previous month. The Pearson correlation coefficient between the DHQ and 3-day dietary records was 0.48 for energy, 0.55 for fat, and 0.48 for carbohydrates among 48 normal-weight women [27]. The DHQ was completed at the baseline, checked by dietitians, and missing or illogical answers were obtained or corrected by interview. To calculate the GI, we estimated the GI according to a strategy used in a previous study with Japanese participants [14]. Briefly, to determine the GI value of each food for use in the calculations, each food item included in the DHQ was directly matched to foods in the international table of GI or in several publications on the GI of Japanese foods [3,30,31]. The dietary GI was calculated by multiplying the contribution of each individual food to the daily available carbohydrate intake using the food’s GI value and then summing the products. The GI values based on 50 grams of available carbohydrates in common Japanese foods and beverages, with glucose used as the reference (GI for glucose = 100), were as follows: GI of white rice = 77, white rice with barley = 67, white rice with germ = 66, brown rice = 55, soba and udon (Japanese noodles) = 47, instant noodles = 47, spaghetti = 46, white bread = 74, oranges = 39, bananas = 51, apples = 37, and soft drinks = 61. Dietary GL was calculated by multiplying the dietary GI by the total amount of daily available carbohydrate intake (divided by 100). For these calculations, a strategy used in previous studies was used and the Pearson correlations between DHQ and dietary records for dietary GI and GL were 0.72 and 0.66 among women and 0.65 and 0.71 among men, respectively [32].

### Data analysis

The characteristics of the study population are presented as the mean or median for continuous variables and as a percentage for categorical variables. We used crude values for dietary GI and energy-adjusted values for dietary GL (/1000 kcal) because, by definition, dietary GI is a measure of carbohydrate quality, not quantity, whereas dietary GL is a measure of the combination of carbohydrate quality and quantity [14]. The LDL levels were calculated using the Friedewald equation: LDL = total cholesterol - (HDL + [TG/5]). Differences in the baseline characteristics among the quartiles were tested using an analysis of variance (ANOVA) for continuous variables and chi-squared tests for categorical variables. We treated each major dietary variable as either continuous or categorical (quartiles) variables.

To investigate the associations of GI and GL with metabolic risk factors, we used a multiple linear regression model to adjust for potential confounding factors including age, sex, visceral fat area, total energy intake, and physical activity levels. We used visceral fat area as a marker of adiposity because prior data suggested that visceral fat plays an important role in the pathogenesis of metabolic disease [33]. Using the BMI or waist circumference instead of the visceral fat area did not result in material differences in the results. We calculated the adjusted-mean HbA1c and FPG; LDL, HDL, and TG concentrations; SBP and DBP; and BMI, waist circumference, visceral fat area, and subcutaneous fat area according to the quartiles of GI and GL. Tests for trends were conducted by assigning the median value to each quartile and modeling this value as a continuous variable. We also examined whether the associations of GI or GL with HbA1c and FPG were modified by sex. The *P*-values for the interaction were calculated by further including the product terms in the regression models using *t*-tests. GL, but not GI, was positively associated with HbA1c in the present study, suggesting that the quantity of carbohydrate may also be associated with HbA1c. Thus, to further examine the associations of carbohydrate intake as well as GI and GL with metabolic risk factors, we generated scatter plots and regression lines and computed Pearson correlation coefficients for all outcome variables. In addition, because there was a suggestive association between GL and poor glycemic control (HbA1c ≥ 7.0% or FPG 150 mg/dL), we conducted a logistic regression analysis to estimate odds ratios and 95% confidence intervals [CIs] for poor glycemic control with adjustment for potential confounding factors including age, sex, visceral fat area, total energy intake, and physical activity levels. Two-sided *P* values <0.05 were considered to be statistically significant. Analyses were carried out using Stata software (version 11; Stata Corp, College Station, TX).

## Results

In total of 227 participants, the participants ranged in age from 40 to 64 years, with a mean age of 54 years. Male participants tended to have a higher GI. Their reported mean total energy intake per day was 2284.0 ± 801.4 kcal. The average dietary GI was 66 ± 5, and the average dietary GL was 79 ± 17 (/1000 kcal). The mean BMI was 30.6 ± 3.1 kg/m^2^, HbA1c was 6.3 ± 1.1%, and FPG was 112 ± 26 mg/dL (6.2 ± 1.4 mmol/L). The baseline characteristics of 227 participants in this study according to the quartiles of GI and GL are shown in Table 1. Participants with high GI tended to have higher GL. Also, participants with high GL tended to have higher GI and higher HbA1c.

**Table 1:** Baseline characteristics according to quartiles of glycemic index and glycemic load

**Quartile of glycemic index**	**Q1**	**Q2**	**Q3**	**Q4**	***P***
Male / Female (n)	24 / 33	25 / 31	26 / 31	38 / 18	0.02
Age (y)	55.3 ± 6.2	54.5 ± 6.4	52.8 ± 6.8	53.9 ± 6.0	0.20
BMI (kg/m^2^)	30.8 ± 3.7	30.5 ± 2.9	30.8 ± 3.0	30.2 ± 2.6	0.74
Waist circumference (cm)	103 ± 9	102 ± 8	103 ± 9	101 ± 6	0.43
Visceral fat area (cm^2^)	143 ± 58	141 ± 46	138 ± 47	150 ± 45	0.58
Subcutaneous fat area (cm^2^)	308 ± 122	285 ± 93	312 ± 99	263 ± 81	0.04
HbA1c (%)	6.2 ± 0.7	6.5 ± 1.4	6.2 ± 1.1	6.2 ± 1.1	0.38
Fasting plasma glucose (mg/dL)	111 ± 23	115 ± 26	111 ± 23	111 ± 31	0.80
Insulin (μIU/mL)	10.9 ± 5.5	13.7 ± 9.8	11.4 ± 10.7	9.9 ± 5.0	0.09
HOMA-IR	3.0 ± 1.7	4.0 ± 3.2	3.2 ± 3.1	2.8 ± 1.6	0.06
HOMA-β	90.2 ± 48.1	108.2 ± 80.7	95.8 ± 95.7	85.3 ± 43.5	0.35
***Daily nutritional intake***					
Energy (kcal/day)	2309 ± 976	2276 ± 718	2177 ± 633	2376 ± 845	0.61
Glycemic index	60 ± 3	65 ± 1	68 ± 1	71 ± 2	<0.001
Glycemic load (/1000 kcal)	67 ± 17	78 ± 13	85 ± 12	89 ± 16	<0.001
**Quartile of glycemic load**	**Q1**	**Q2**	**Q3**	**Q4**	***P***
Male / Female (n)	33 / 24	24 / 32	27 / 30	29 / 27	0.43
Age (y)	53.4 ± 6.6	54.3 ± 6.4	54.5 ± 6.1	54.3 ± 6.5	0.79
BMI (kg/m^2^)	30.7 ± 3.6	30.1 ± 2.9	30.5 ± 2.7	30.9 ± 3.0	0.55
Waist circumference (cm)	103 ± 9	101 ± 8	102 ± 7	103 ± 8	0.64
Visceral fat area (cm^2^)	143 ± 57	137 ± 44	144 ± 49	148 ± 47	0.73
Subcutaneous fat area (cm^2^)	294 ± 119	290 ± 101	293 ± 88	291 ± 96	>0.99
HbA1c (%)	6.1 ± 0.6	6.1 ± 1.0	6.5 ± 1.3	6.4 ± 1.3	0.05
Fasting plasma glucose (mg/dL)	110 ± 16	106 ± 16	119 ± 34	112 ± 31	0.06
Insulin (μIU/mL)	11.2 ± 5.9	11.0 ± 8.5	12.1 ±10.9	11.6 ± 6.9	0.89
HOMA-IR	3.1 ± 1.8	3.0 ± 2.7	3.5 ± 3.0	3.4 ± 2.5	0.66
HOMA-β	92.6 ± 53.4	94.3 ± 61.6	94.2 ± 92.0	98.5 ± 71.0	0.98
***Daily nutritional intake***					
Energy (kcal/day)	2542 ± 893	2304 ± 773	2245 ± 664	2040 ± 798	0.01
Glycemic index	63 ± 5	66 ± 4	67 ± 3	69 ± 3	<0.001
Glycemic load (/1000 kcal)	58 ± 11	75 ± 3	86 ± 3	100 ± 9	<0.001

Data are (n) or means ± SD. Differences in baseline characteristics between quartiles were tested using analysis of variance (ANOVA) for categorical variables and chi-squared tests for continuous variables.

**Figure 1:**
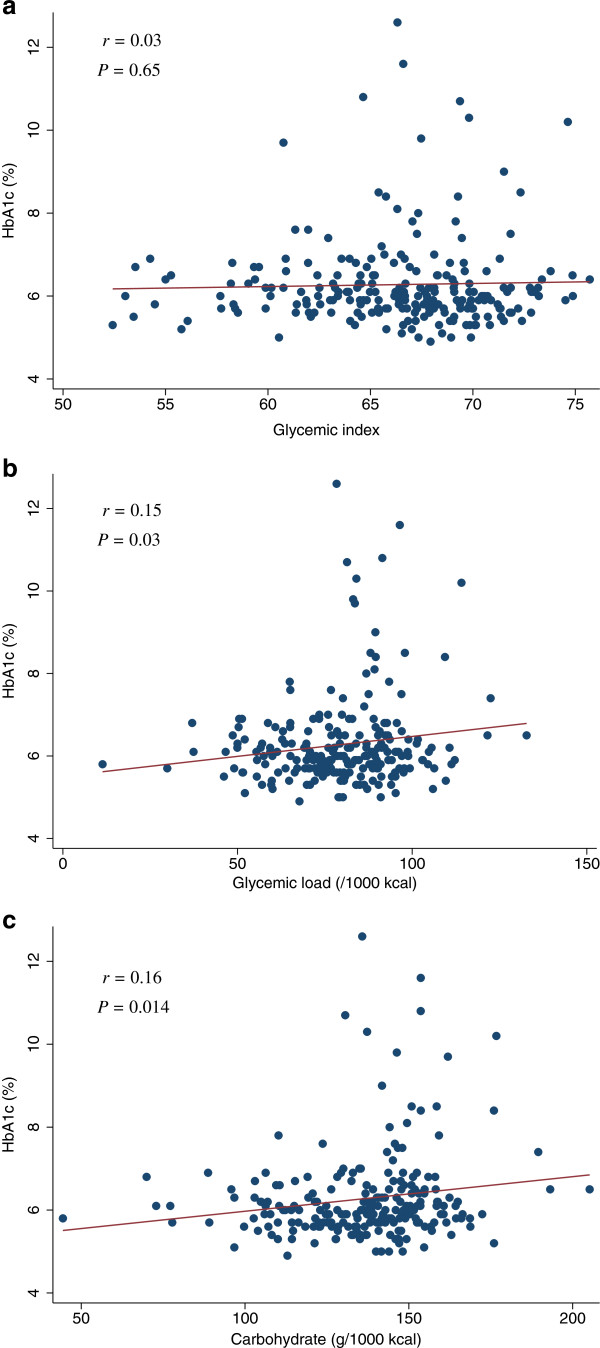
**Scatter plots with regression lines of glycemic index, glycemic load, and carbohydrate intake against HbA1c.** Pearson correlation coefficients (*r*) and corresponding *P*-values are shown. X-axis: glycemic index (Figure 1a), glycemic load (/1000 kcal) (Figure 1b), and carbohydrate intake (g/1000 kcal) (Figure 1c). Y-axis: HbA1c.

**Figure 2:**
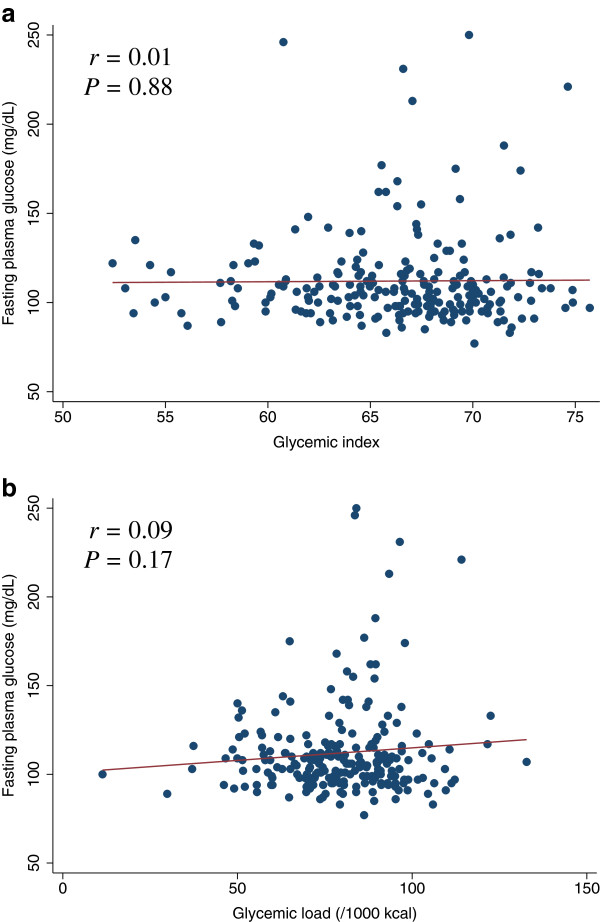
**Scatter plots with regression lines of glycemic index and glycemic load against fasting plasma glucose.** Pearson correlation coefficients (*r*) and corresponding *P*-values are shown. X-axis: glycemic index (Figure 2a) and glycemic load (/1000 kcal) (Figure 2b). Y-axis: Fating plasma glucose.

Table 2 shows associations of GI and GL with FPG, HbA1c, HOMA-IR, and HOMA-β. GL, but not GI, was positively associated with HbA1c (Table 2 and Figure 1). For increasing quartiles of GI, the corresponding adjusted mean HbA1c levels were 6.3%, 6.7%, 6.4%, and 6.4% (*P* for trend = 0.991). The scatter plot of GL (/1000 kcal) against HbA1c indicated a positive linear relation between GL and HbA1c (*r* = 0.15, *P* = 0.026) (Figure 1b). Among participants with HbA1c ≥ 7.0%, 20 out of 28 (71%) had a high GL (≥ median) and the adjusted odds ratio for HbA1c ≥ 7.0% among participants with a higher GL was 3.1 (95% confidence interval [CI] = 1.2 to 8.1) compared to the participants with a lower GL (<median). In addition, a multiple linear regression analysis suggested a positive association between GL and HbA1c. For increasing quartiles of GL, the corresponding adjusted mean HbA1c were 6.2%, 6.2%, 6.6%, and 6.5% (*P* for trend = 0.044) (Table 2). Also, the associations of GI or GL with HbA1c and FPG were not modified by sex (*P* for interaction >0.10). We further examined the association of carbohydrate intake with HbA1c. The scatter plots of carbohydrate intake (g/1000 kcal) against HbA1c indicated a positive linear relation between carbohydrate intake and HbA1c (*r* = 0.16, *P* = 0.014) (Figure 1c). After adjustments for potential confounders, carbohydrate intake was also positively associated with HbA1c. For increasing quartiles of carbohydrate intake, the corresponding adjusted mean HbA1c levels were 6.1%, 6.3%, 6.3%, and 6.6% (*P* for trend = 0.026) (data not shown). Although GL was not linearly related to FPG, individuals with high FPG (≥ 150 mg/dL) tended to have a high GL (Figure 2b). Among 16 participants with FPG ≥ 150 mg/dL, 13 participants (81.3%) had a higher GL (Figure 2b) and the adjusted odds ratio for FPG ≥ 150 mg/dL among participants with higher GL values was 8.5 (95% confidence interval = 1.7 to 43.4) compared to the lower GL group.

**Table 2:** **HbA1c, fasting plasma glucose, HOMA-IR, and HOMA-β according to dietary glycemic index and energy-adjusted dietary glycemic load**^**1**^

**Dietary variables**	***n***	**HbA1c %**	*P *for trend*	**Fasting plasma glucose *mg/dL***	*P *for trend*	**HOMA-IR**	*P *for trend*	**HOMA-β**	*P *for trend*
Quartile of glycemic index ^2^									
Q1 [60]	57	6.3 (5.7, 6.9)	0.991	116 (101, 131)	0.900	3.0 (1.7, 4.3)	0.379	77 (37, 116)	0.392
Q2 [65]	57	6.7 (6.0, 7.3)		120 (105, 136)		3.7 (2.4, 5.0)		90 (49, 132)	
Q3 [68]	57	6.4 (5.8, 7.0)		117 (102, 132)		3.1 (1.8, 4.4)		78 (38, 118)	
Q4 [71]	56	6.4 (5.8, 6.9)		117 (103, 131)		2.6 (1.4, 3.8)		66 (28, 103)	
Quartile of glycemic load ^2^									
Q1 [58]	57	6.2 (5.5, 6.8)	0.044	116 (101, 131)	0.322	2.7 (1.3, 4.0)	0.250	65 (24, 105)	0.428
Q2 [75]	57	6.2 (5.5, 6.8)		111 (97, 126)		2.4 (1.1, 3.7)		68 (28, 109)	
Q3 [86]	57	6.6 (6.0, 7.2)		124 (110, 139)		3.1 (1.8, 4.5)		70 (30, 110)	
Q4 [100]	56	6.5 (5.9, 7.1)		117 (103, 130)		3.0 (1.8, 4.2)		76 (38, 113)	

Abbreviations: HOMA-IR, homeostatic model assessment for insulin resistance; HOMA-β, homeostasis model assessment for β-cell function.

^1^ Adjusted mean levels and 95% CI in parentheses. Glycemic load was defined as an indicator of blood glucose induced by an individual’s total available carbohydrate.

^2^ Median value for each quartile in brackets. *A multiple linear regression model was used to adjust for potential confounding factors including age, sex, visceral fat area, total energy intake, and physical activity level.

**Figure 3:**
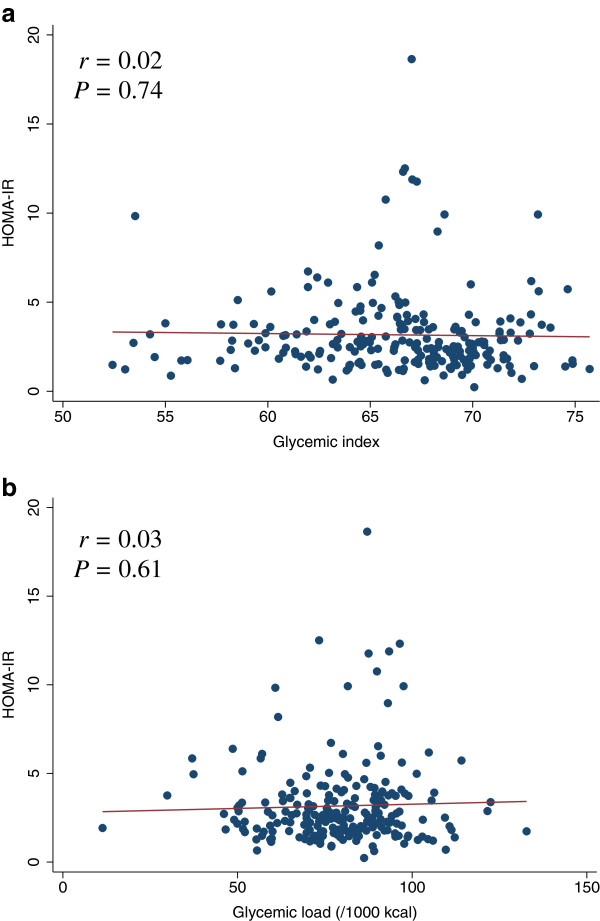
**Scatter plots with regression lines of glycemic index and glycemic load against HOMA-IR**. Pearson correlation coefficients (*r*) and corresponding *P*-values are shown. X-axis: glycemic index (Figure 3a) and glycemic load (/1000 kcal) (Figure 3b). Y-axis: HOMA-IR.

**Figure 4:**
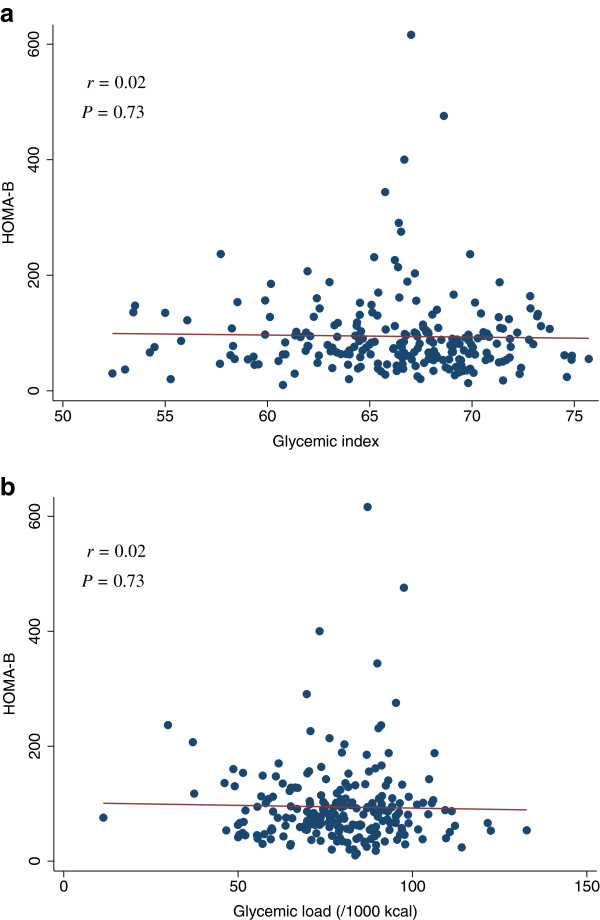
**Scatter plots with regression lines of glycemic index and glycemic load against HOMA-β.** Pearson correlation coefficients (*r*) and corresponding *P*-values are shown. X-axis: glycemic index (Figure 4a) and glycemic load (/1000 kcal) (Figure 4b). Y-axis: HOMA-β.

**Figure 5:**
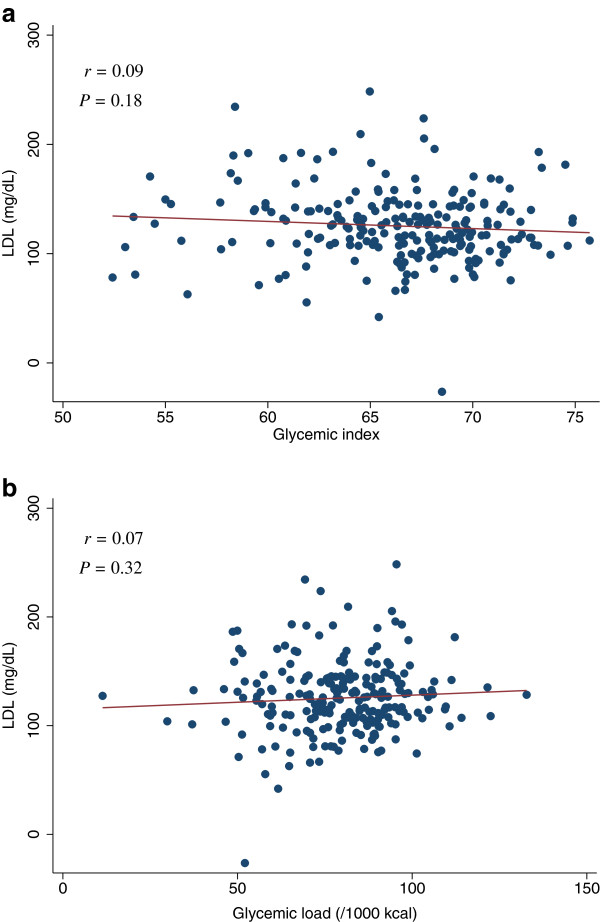
**Scatter plots with regression lines of glycemic index and glycemic load against LDL.** Pearson correlation coefficients (*r*) and corresponding *P*-values are shown. X-axis: glycemic index (Figure 5a) and glycemic load (/1000 kcal) (Figure 5b). Y-axis: LDL.

**Figure 6:**
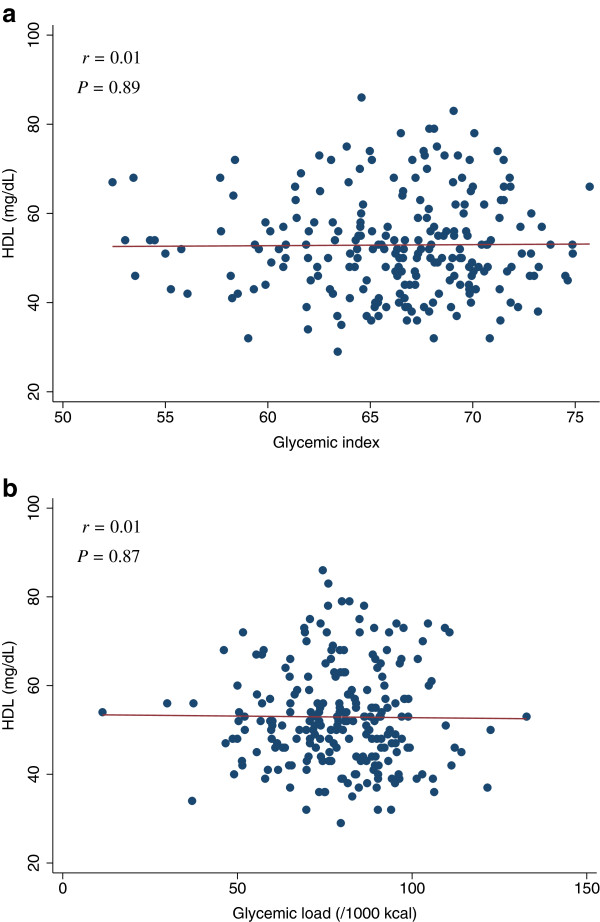
**Scatter plots with regression lines of glycemic index and glycemic load against HDL.** Pearson correlation coefficients (*r*) and corresponding *P*-values are shown. X-axis: glycemic index (Figure 6a) and glycemic load (/1000 kcal) (Figure 6b). Y-axis: HDL.

**Figure 7:**
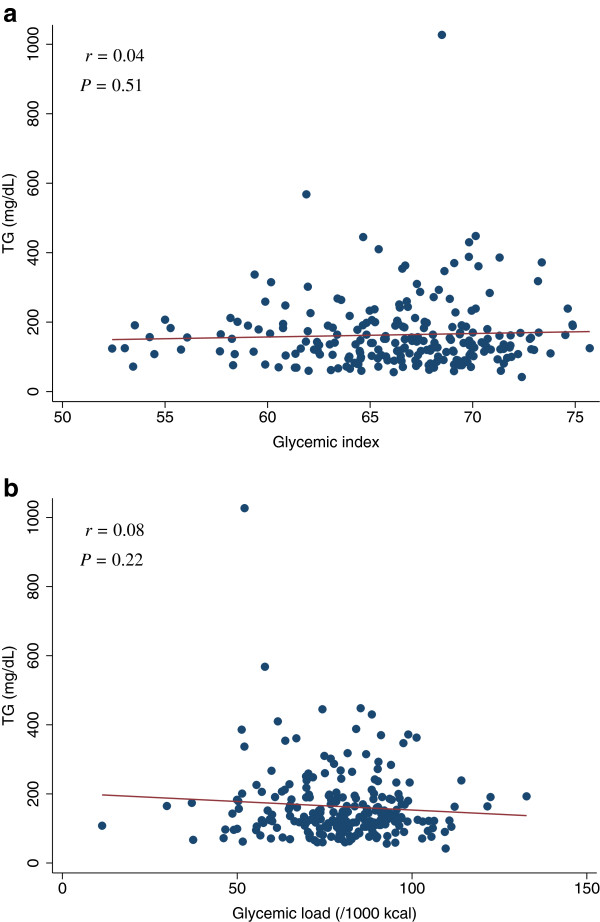
**Scatter plots with regression lines of glycemic index and glycemic load against TG.** Pearson correlation coefficients (*r*) and corresponding *P*-values are shown. X-axis: glycemic index (Figure 7a) and glycemic load (/1000 kcal) (Figure 7b). Y-axis: TG.

**Figure 8:**
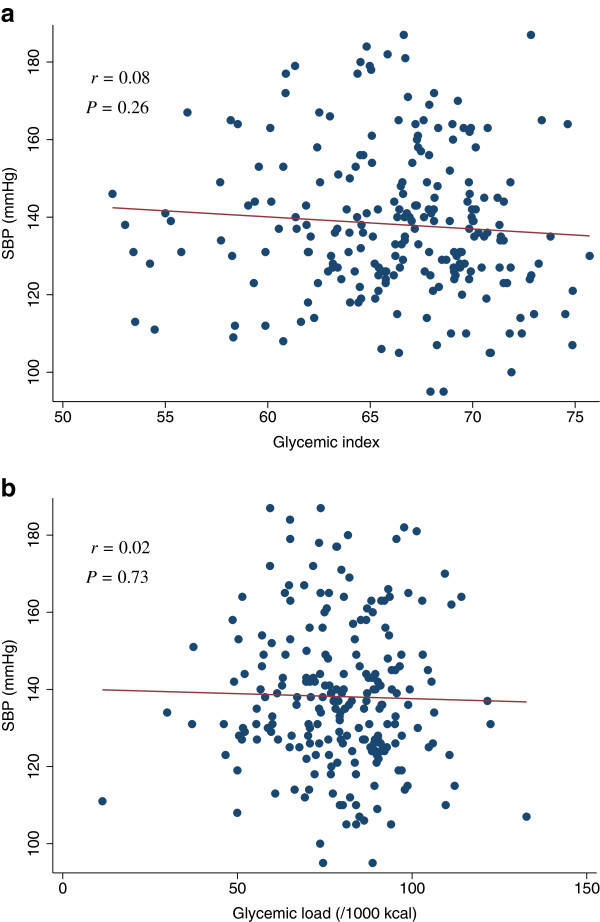
**Scatter plots with regression lines of glycemic index and glycemic load against SBP.** Pearson correlation coefficients (*r*) and corresponding *P*-values are shown. X-axis: glycemic index (Figure 8a) and glycemic load (/1000 kcal) (Figure 8b). Y-axis: SBP.

**Figure 9:**
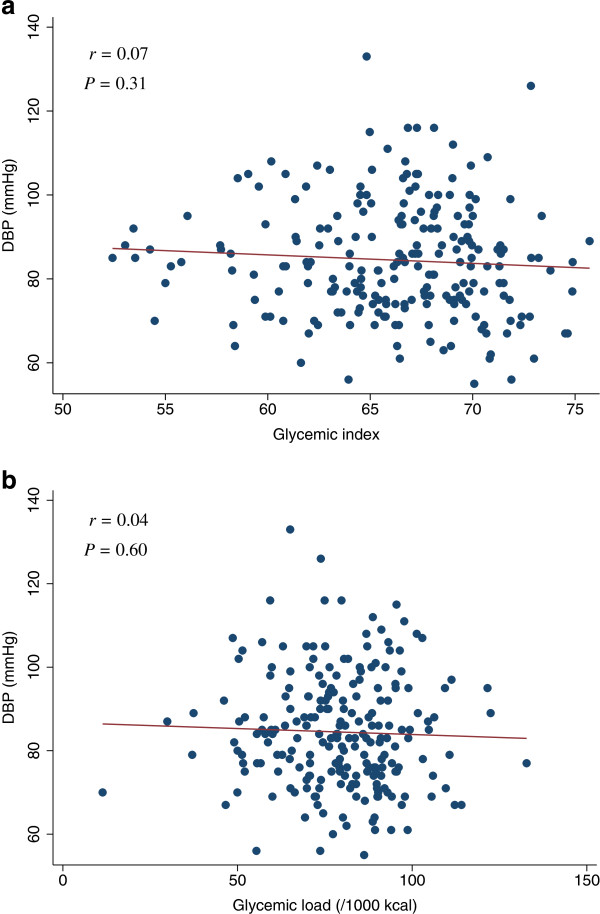
**Scatter plots with regression lines of glycemic index and glycemic load against DBP.** Pearson correlation coefficients (*r*) and corresponding *P*-values are shown. X-axis: glycemic index (Figure 9a) and glycemic load (/1000 kcal) (Figure 9b). Y-axis: DBP.

**Figure 10:**
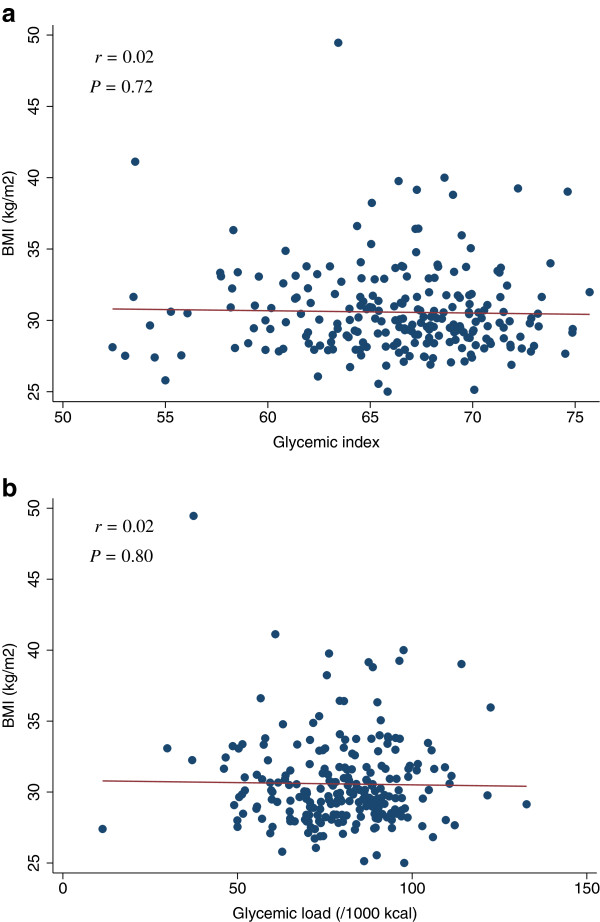
**Scatter plots with regression lines of glycemic index and glycemic load against BMI.** Pearson correlation coefficients (*r*) and corresponding *P*-values are shown. X-axis: glycemic index (Figure 10a) and glycemic load (/1000 kcal) (Figure 10b). Y-axis: BMI.

**Figure 11:**
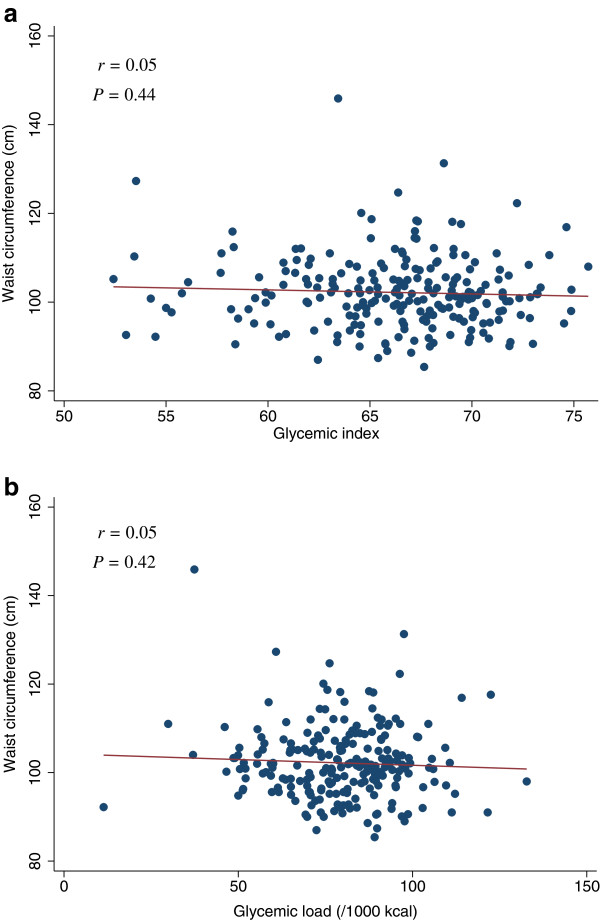
**Scatter plots with regression lines of glycemic index and glycemic load against waist circumference.** Pearson correlation coefficients (*r*) and corresponding *P*-values are shown. X-axis: glycemic index (Figure 11a) and glycemic load (/1000 kcal) (Figure 11b). Y-axis: Waist circumference.

**Figure 12:**
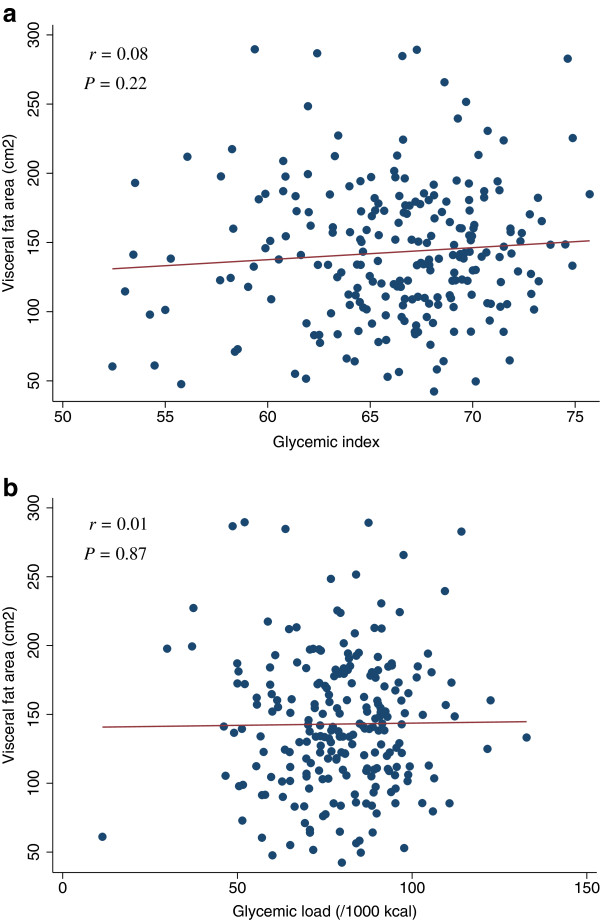
**Scatter plots with regression lines of glycemic index and glycemic load against visceral fat area.** Pearson correlation coefficients (*r*) and corresponding *P*-values are shown. X-axis: glycemic index (Figure 12a) and glycemic load (/1000 kcal) (Figure 12b). Y-axis: Visceral fat area.

**Figure 13:**
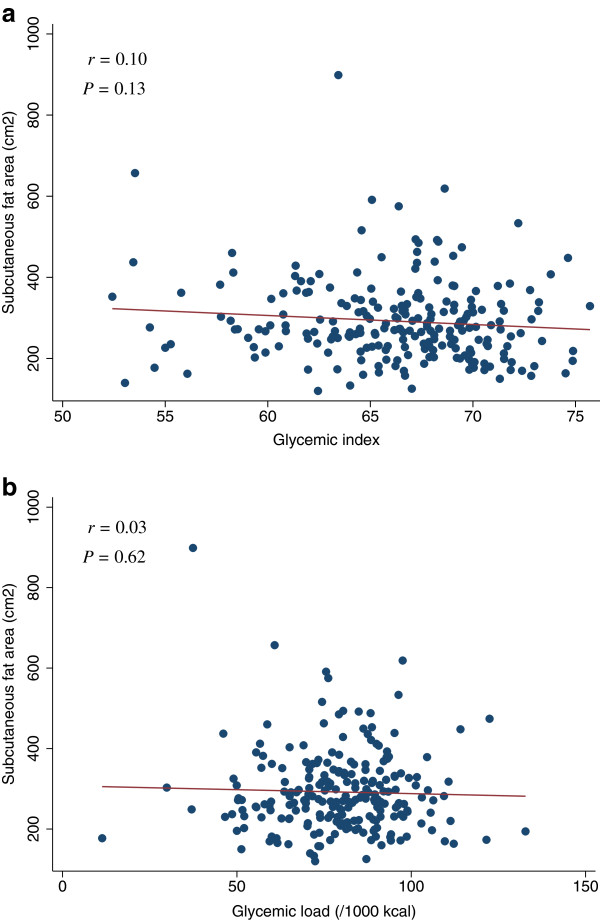
**Scatter plots with regression lines of glycemic index and glycemic load against subcutaneous fat area.** Pearson correlation coefficients (*r*) and corresponding *P*-values are shown. X-axis: glycemic index (Figure 13a) and glycemic load (/1000 kcal) (Figure 13b). Y-axis: Subcutaneous fat area.

After adjustments for potential confounding factors, the dietary GI or GL was not associated with FPG, HOMA-IR, or HOMA-β (Table 2). Also, after adjustments for potential confounding factors, the GI or GL was not associated with the lipid levels (LDL, HDL, or TG), SBP, or DBP (Table 3). In the analysis of the adiposity measures (BMI, waist, visceral fat area, or subcutaneous fat area), GI or GL was not associated with the adiposity measures after adjustments for potential confounding factors (Table 4). The scatter plots of GI and GL (/1000 kcal) against HOMA-IR, HOMA-β, lipid levels (LDL, HDL, or TG), SBP, or DBP did not indicate any apparent associations between these measures (Figures 3, 4, 5, 6, 7, 8, 9, 10, 11, 12, 13).

**Table 3:** **Lipids and blood pressure according to dietary glycemic index and energy-adjusted dietary glycemic load**^**1**^

**Dietary variables**	***n***	**LDL *mg/dL ***	*P *for trend*	**HDL *mg/dL ***	*P *for trend*	**TG *mg/dL ***	*P *for trend*	**SBP *mmHg ***	*P *for trend*	**DBP *mmHg ***	*P *for trend*
Quartile of glycemic index ^2^											
Q1 [60]	57	116 (97, 134)	0.457	45 (39, 52)	0.195	156 (98, 214)	0.987	125 (115, 136)	0.686	78 (70, 85)	0.677
Q2 [65]	57	109 (89, 128)		44 (38, 51)		150 (90, 211)		129 (118, 140)		79 (71, 87)	
Q3 [68]	57	108 (89, 126)		48 (42, 54)		158 (99, 217)		128 (117, 138)		81 (73, 88)	
Q4 [71]	56	112 (95, 130)		47 (42, 53)		154 (100, 209)		124 (113, 134)		75 (68, 83)	
Quartile of glycemic load^2^											
Q1 [58]	57	111 (92, 130)	0.579	47 (40, 53)	0.831	179 (120, 238)	0.171	127 (116, 138)	0.387	78 (70, 86)	0.509
Q2 [75]	57	105 (86, 124)		48 (42, 54)		148 (90, 206)		125 (114, 136)		78 (71, 86)	
Q3 [86]	57	109 (90, 127)		46 (39, 52)		155 (96, 214)		120 (109, 130)		75 (67, 83)	
Q4 [100]	56	115 (98, 133)		47 (41, 53)		151 (96, 205)		126 (116, 136)		78 (70, 85)	

^1^ Adjusted mean levels and 95% CI in parentheses. Glycemic load was defined as an indicator of blood glucose induced by an individual’s total available carbohydrate.

^2^ Median value for each quartile in brackets. *A multiple linear regression model was used to adjust for potential confounding factors including age, sex, visceral fat area, total energy intake, and physical activity level.

**Table 4:** **BMI, waist, and fat area according to dietary glycemic index and energy-adjusted dietary glycemic load**^**1**^

**Dietary variables**	***n***	**BMI *kg/m***^***2***^	*P *for trend*	**Waist circumference *cm***	*P *for trend*	**Visceral fat area *cm***^***2***^	*P *for trend*	**Subcutaneous fat area *cm***^***2***^	*P *for trend*
Quartile of glycemic index^2^									
Q1 [60]	57	30.0 (28.3, 31.7)	0.378	101 (97, 106)	0.281	176 (149, 203)	0.966	219 (168, 270)	0.257
Q2 [65]	57	29.6 (27.9, 31.4)		100 (95, 105)		174 (146, 202)		195 (143, 248)	
Q3 [68]	57	29.7 (28.0, 31.4)		101 (96, 105)		172 (145, 200)		220 (169, 272)	
Q4 [71]	56	29.4 (27.8, 31.1)		99 (95, 104)		177 (151, 202)		191 (143, 239)	
Quartile of glycemic load ^2^									
Q1 [58]	57	29.8 (28.0, 31.5)	0.924	101 (96, 105)	0.754	173 (145, 200)	0.477	213 (161, 266)	0.590
Q2 [75]	57	29.1 (27.4, 30.8)		99 (94, 103)		169 (142, 197)		197 (145, 248)	
Q3 [86]	57	29.4 (27.7, 31.2)		99 (95, 104)		176 (149, 203)		204 (152, 256)	
Q4 [100]	56	29.8 (28.2, 31.4)		100 (96, 105)		179 (153, 204)		203 (155, 251)	

^1^ Adjusted mean levels and 95% CI in parentheses. Glycemic load was defined as an indicator of blood glucose induced by an individual’s total available carbohydrate.

^2^ Median value for each quartile in brackets. *A multiple linear regression model was used to adjust for potential confounding factors including age, sex, total energy intake, and physical activity level.

## Discussion

In this study of 227 obese Japanese participants, participants with poor glycemic control tended to have a higher GL, whereas GI was not associated with glycemia. No association between GI or GL and the BMI, waist circumference, visceral fat area, subcutaneous fat area, LDL, HDL, TG, SBP, or DBP was observed.

In preceding studies, an increased diabetes risk was reportedly associated with GI and GL [6,7,34,35], while other studies reported no association [8-10]. Blood glucose levels are effectively controlled, unless there is a dysregulation of glucose metabolism [36], a condition observed in individuals with diabetes, prediabetes, or metabolic syndrome. Therefore, the findings in preceding studies may be inconclusive in part because in some studies, there might have been few participants with abnormal glucose metabolism at baseline or during follow-up periods. In our study, GL showed a positive relation with HbA1c. In fact, among 28 participants with HbA1c ≥7.0%, 20 participants (71%) had a higher GL. Nonetheless, longer duration of observation is necessary to confirm the findings. Also, in a multiple linear regression analysis, we observed no association between GI or GL and FPG. Indices reflecting both fasting and postprandial hyperglycemia such as HbA1c may have shown stronger association for GL, because GL may play major roles in postprandial hyperglycemia. Also, the within-subject variability of HbA1c is smaller than FPG, and this may account for the stronger association of GL with HbA1c than FPG [37]. Thus, we possibly lacked statistical power to detect a linear association between GL and FPG. Indeed, when we dichotomized FPG and GL, individuals with high FPG (≥ 150 mg/dL) tended to have a high GL; among 16 participants with FPG ≥ 150 mg/dL, 13 participants (81.3%) had a high GL (≥ median). These findings suggest that a high GL is associated with poor glycemic control. GI has been reported to be positively associated with insulin resistance and metabolic syndrome [38], and in one study, it was shown that although a high GI predicted the risk of type 2 diabetes among non-abdominally obese individuals, no association was observed in abdominally obese individuals [9]. Since the participants of our study were obese, with a mean visceral fat area of 143.0 cm^2^, this may have masked the relationship between GI and HbA1c or FPG levels in our study. Also, the dietary GI has been reported not to reflect the total carbohydrate intake, and may provide minimal insight into the overall insulin demand induced by the total carbohydrate intake [7,35]. Also, studies reported that a high dietary GL was associated with other health outcomes, such as dyslipidemia and coronary heart disease [39]. In a preceding study, GI and GL were reported to be positively related to the risk of metabolic syndrome among women with a BMI ≥25 kg/m^2^[40]. GI and GL have also been reported to be inversely related to the HDL level and positively related to the TG level [39]. However, in our study, no associations between GI or GL and such metabolic risk factors were observed.

In the current study, GL was positively related with HbA1c, while there was no association between GI and HbA1c, suggesting that the amount of the carbohydrate intake may also be associated with HbA1c. Thus, we also investigated the relation between carbohydrate intake and HbA1c. There was a significant relation between carbohydrate intake and HbA1c (*r* = 0.16), and the correlation was similar to the relation between GL and HbA1c (*r* = 0.15). This might suggest that the association of GL with HbA1c might have been mainly driven by carbohydrate content. Of note, it has been reported that carbohydrate restriction improves glycemic control [41,42], suggesting the role of carbohydrate content in glycemic control. However, GL has been found to be a more powerful predictor of postprandial glycemia and insulinemia than was the available carbohydrate content [43]. In addition, several prospective cohort studies have associated GL, but not carbohydrate, with risks of coronary heart disease and type 2 diabetes [35,39]. Taken together, the superiority of carbohydrate amount or GL in glycemic control still remains an open research question.

Some limitations of the present study need to be addressed. First, the DHQ was not specifically designed to measure GI and GL, and the dietary records of obese people have been reported to be inaccurate [44]. More specifically, obese people tend to underreport their caloric intake [45]. Thus, the dietary GI and GL estimated using the DHQ might have been more inaccurate than that estimated by healthy individuals with a normal BMI. However, the bias introduced by underreporting and the measurement error tends to be non-differential with respect to the outcomes, which tends to attenuate the association. Moreover, because total energy adjustments have been reported to potentially reduce the bias caused by underreporting [46-48], we have adjusted for the total energy intake in all the models. Also, a validation study showed a good agreement between the DHQ and the dietary records for GI and GL [32]. Second, the participants in this study were obese adults who were recruited from members who visited health checkups. Thus, selection bias and generalizability may be a problem. Third, dietary intakes were measured only once, and thus may not have reflected the long-term intake. Fourth, as is always the case in observational studies, there may be residual confounding by unknown or unmeasured confounding factors. However, our multiple sensitivity analyses controlling for covariates including visceral fat area, subcutaneous fat area, BMI, and waist circumference showed a positive association between the GL and HbA1c. Finally, it should be mentioned that, using a cross-sectional analysis, we are unable to establish a temporal relationship in the association between GI or GL and metabolic risk factors and given the multiple testing and the limited power of the study, the association between GL and HbA1c needs to be interpreted cautiously.

## Conclusions

Although our results do not answer questions concerning the differential roles of GL and total carbohydrate intakes in the glucose metabolism, our findings suggest that participants with poor glycemic control tend to have a higher GL in an obese Japanese population.

## Competing interests

The authors declare that they have no competing interests.

## Authors’ contributions

MG researched the data, contributed to the discussion, and wrote the manuscript. AM and AG researched the data, contributed to the discussion, and reviewed / edited the manuscript. TS and YT contributed to the discussion. NA, MM, SS, MN, and SW contributed to the discussion, and reviewed / edited the manuscript. All authors read and approved the final manuscript.

## References

[B1] Wild S Roglic G Green A Sicree R King H Global prevalence of diabetes: estimates for the year 2000 and projections for 2030 Diabetes Care 2004 27 1047 1053 10.2337/diacare.27.5.1047 15111519

[B2] Jenkins DJ Wolever TM Taylor RH Barker H Fielden H Baldwin JM Bowling AC Newman HC Jenkins AL Goff DV Glycemic index of foods: a physiological basis for carbohydrate exchange Am J Clin Nutr 1981 34 362 366 6259925 10.1093/ajcn/34.3.362

[B3] Foster-Powell K Holt SH Brand-Miller JC International table of glycemic index and glycemic load values: 2002 Am J Clin Nutr 2002 76 5 56 12081815 10.1093/ajcn/76.1.5

[B4] Sakurai M Nakamura K Miura K Takamura T Yoshita K Morikawa Y Ishizaki M Kido T Naruse Y Suwazono Y Dietary glycemic index and risk of type 2 diabetes mellitus in middle-aged Japanese men Metabolism 2012 61 47 55 10.1016/j.metabol.2011.05.015 21803381

[B5] Villegas R Liu S Gao YT Yang G Li H Zheng W Shu XO Prospective study of dietary carbohydrates, glycemic index, glycemic load, and incidence of type 2 diabetes mellitus in middle-aged Chinese women Arch Intern Med 2007 167 2310 2316 10.1001/archinte.167.21.2310 18039989

[B6] Schulze MB Liu S Rimm EB Manson JE Willett WC Hu FB Glycemic index, glycemic load, and dietary fiber intake and incidence of type 2 diabetes in younger and middle-aged women Am J Clin Nutr 2004 80 348 356 15277155 10.1093/ajcn/80.2.348

[B7] Salmeron J Ascherio A Rimm EB Colditz GA Spiegelman D Jenkins DJ Stampfer MJ Wing AL Willett WC Dietary fiber, glycemic load, and risk of NIDDM in men Diabetes Care 1997 20 545 550 10.2337/diacare.20.4.545 9096978

[B8] Mosdol A Witte DR Frost G Marmot MG Brunner EJ Dietary glycemic index and glycemic load are associated with high-density-lipoprotein cholesterol at baseline but not with increased risk of diabetes in the Whitehall II study Am J Clin Nutr 2007 86 988 994 17921375 10.1093/ajcn/86.4.988

[B9] Schulz M Liese AD Fang F Gilliard TS Karter AJ Is the association between dietary glycemic index and type 2 diabetes modified by waist circumference? Diabetes Care 2006 29 1102 1104 10.2337/dc06-0056 16644644

[B10] Meyer KA Kushi LH Jacobs DR Jr Slavin J Sellers TA Folsom AR Carbohydrates, dietary fiber, and incident type 2 diabetes in older women Am J Clin Nutr 2000 71 921 930 10731498 10.1093/ajcn/71.4.921

[B11] Woo J Ho SC Sham A Sea MM Lam KS Lam TH Janus ED Diet and glucose tolerance in a Chinese population Eur J Clin Nutr 2003 57 523 530 10.1038/sj.ejcn.1601586 12700613

[B12] Hu EA Pan A Malik V Sun Q White rice consumption and risk of type 2 diabetes: meta-analysis and systematic review BMJ 2012 344 e1454 10.1136/bmj.e1454 22422870 PMC3307808

[B13] Nanri A Mizoue T Noda M Takahashi Y Kato M Inoue M Tsugane S Rice intake and type 2 diabetes in Japanese men and women: the Japan Public Health Center-based Prospective Study Am J Clin Nutr 2010 92 1468 1477 10.3945/ajcn.2010.29512 20980490

[B14] Murakami K Sasaki S Takahashi Y Okubo H Hosoi Y Horiguchi H Oguma E Kayama F Dietary glycemic index and load in relation to metabolic risk factors in Japanese female farmers with traditional dietary habits Am J Clin Nutr 2006 83 1161 1169 16685061 10.1093/ajcn/83.5.1161

[B15] Sartorelli DS Freire RD Ferreira SR Cardoso MA Dietary fiber and glucose tolerance in Japanese Brazilians Diabetes Care 2005 28 2240 2242 10.2337/diacare.28.9.2240 16123498

[B16] Sartorelli DS Franco LJ Gimeno SG Ferreira SR Cardoso MA Dietary fructose, fruits, fruit juices and glucose tolerance status in Japanese-Brazilians Nutr Metabol Cardiovasc Dis: NMCD 2009 19 77 83 10.1016/j.numecd.2008.04.004 18676134

[B17] Watanabe S Morita A Aiba N Miyachi M Sasaki S Morioka M Noda M Takebayashi T Kimura M Study Design of the Saku Control Obesity Program (SCOP) Anti Aging Med 2007 4 70 73 10.3793/jaam.4.70

[B18] Morita A Ohmori Y Suzuki N Ide N Morioka M Aiba N Sasaki S Miyachi M Noda M Watanabe S Anthropometric and Clinical Findings in Obese Japanese: The Saku Control Obesity Program (SCOP) Anti Aging Med 2008 5 13 16 10.3793/jaam.5.13

[B19] Tanaka T Morita A Kato M Hirai T Mizoue T Terauchi Y Watanabe S Noda M Group SS Congener-specific polychlorinated biphenyls and the prevalence of diabetes in the Saku Control Obesity Program (SCOP) Endocr J 2011 58 589 596 10.1507/endocrj.K10E-361 21551956

[B20] White WB Anwar YA Evaluation of the overall efficacy of the Omron office digital blood pressure HEM-907 monitor in adults Blood Press Monit 2001 6 107 110 10.1097/00126097-200104000-00007 11433132

[B21] Yoshizumi T Nakamura T Yamane M Islam AH Menju M Yamasaki K Arai T Kotani K Funahashi T Yamashita S Matsuzawa Y Abdominal fat: standardized technique for measurement at CT Radiology 1999 211 283 286 10189485 10.1148/radiology.211.1.r99ap15283

[B22] Oka R Miura K Sakurai M Nakamura K Yagi K Miyamoto S Moriuchi T Mabuchi H Yamagishi M Takeda Y Comparison of waist circumference with body mass index for predicting abdominal adipose tissue Diabetes Res Clin Pract 2009 83 100 105 10.1016/j.diabres.2008.10.001 19019478

[B23] Seino Y Nanjo K Tajima N Kadowaki T Kashiwagi A Araki E Ito C Inagaki N Iwamoto Y Kasuga M Report of the Committee on the classification and diagnostic criteria of diabetes mellitus Diabetol Int 2010 1 2 20 10.1007/s13340-010-0006-7 PMC4020724 24843435

[B24] Matthews DR Hosker JP Rudenski AS Naylor BA Treacher DF Turner RC Homeostasis model assessment: insulin resistance and beta-cell function from fasting plasma glucose and insulin concentrations in man Diabetologia 1985 28 412 419 10.1007/BF00280883 3899825

[B25] Wallace TM Levy JC Matthews DR Use and abuse of HOMA modeling Diabetes Care 2004 27 1487 1495 10.2337/diacare.27.6.1487 15161807

[B26] Sasaki S Ushio F Amano K Morihara M Todoriki O Uehara Y Toyooka E Serum biomarker-based validation of a self-administered diet history questionnaire for Japanese subjects J Nutr Sci Vitaminol (Tokyo) 2000 46 285 296 10.3177/jnsv.46.285 11227800

[B27] Sasaki S Yanagibori R Amano K Self-administered diet history questionnaire developed for health education: a relative validation of the test-version by comparison with 3-day diet record in women J Epidemiol 1998 8 203 215 10.2188/jea.8.203 9816812

[B28] Sasaki S Yanagibori R Amano K Validity of a self-administered diet history questionnaire for assessment of sodium and potassium: comparison with single 24-hour urinary excretion Jpn Circ J 1998 62 431 435 10.1253/jcj.62.431 9652319

[B29] Okubo H Sasaki S Rafamantanantsoa HH Ishikawa-Takata K Okazaki H Tabata I Validation of self-reported energy intake by a self-administered diet history questionnaire using the doubly labeled water method in 140 Japanese adults Eur J Clin Nutr 2008 62 1343 1350 10.1038/sj.ejcn.1602858 17671444

[B30] Sugiyama M Tang AC Wakaki Y Koyama W Glycemic index of single and mixed meal foods among common Japanese foods with white rice as a reference food Eur J Clin Nutr 2003 57 743 752 10.1038/sj.ejcn.1601606 12792658

[B31] Fernandes G Velangi A Wolever TM Glycemic index of potatoes commonly consumed in North America J Am Diet Assoc 2005 105 557 562 10.1016/j.jada.2005.01.003 15800557

[B32] Murakami K Sasaki S Takahashi Y Okubo H Hirota N Notsu A Fukui M Date C Reproducibility and relative validity of dietary glycaemic index and load assessed with a self-administered diet-history questionnaire in Japanese adults Br J Nutr 2008 99 639 648 17764595 10.1017/S0007114507812086

[B33] Matsushita Y Nakagawa T Yamamoto S Takahashi Y Yokoyama T Noda M Mizoue T Associations of visceral and subcutaneous fat areas with the prevalence of metabolic risk factor clustering in 6,292 Japanese individuals: the Hitachi Health Study Diabetes Care 2010 33 2117 2119 10.2337/dc10-0120 20460443 PMC2928375

[B34] Hodge AM English DR O'Dea K Giles GG Glycemic index and dietary fiber and the risk of type 2 diabetes Diabetes Care 2004 27 2701 2706 10.2337/diacare.27.11.2701 15505008

[B35] Salmeron J Manson JE Stampfer MJ Colditz GA Wing AL Willett WC Dietary fiber, glycemic load, and risk of non-insulin-dependent diabetes mellitus in women JAMA 1997 277 472 477 10.1001/jama.1997.03540300040031 9020271

[B36] Aronoff SL Berkowitz K Shreiner B Want L Glucose Metabolism and Regulation: Beyond Insulin and Glucagon Diabetes Spectrum 2004 17 183 190 10.2337/diaspect.17.3.183

[B37] Rohlfing CL Wiedmeyer HM Little RR England JD Tennill A Goldstein DE Defining the relationship between plasma glucose and HbA(1c): analysis of glucose profiles and HbA(1c) in the Diabetes Control and Complications Trial Diabetes Care 2002 25 275 278 10.2337/diacare.25.2.275 11815495

[B38] McKeown NM Meigs JB Liu S Saltzman E Wilson PW Jacques PF Carbohydrate nutrition, insulin resistance, and the prevalence of the metabolic syndrome in the Framingham Offspring Cohort Diabetes Care 2004 27 538 546 10.2337/diacare.27.2.538 14747241

[B39] Liu S Manson JE Stampfer MJ Holmes MD Hu FB Hankinson SE Willett WC Dietary glycemic load assessed by food-frequency questionnaire in relation to plasma high-density-lipoprotein cholesterol and fasting plasma triacylglycerols in postmenopausal women Am J Clin Nutr 2001 73 560 566 11237932 10.1093/ajcn/73.3.560

[B40] Kim K Yun SH Choi BY Kim MK Cross-sectional relationship between dietary carbohydrate, glycaemic index, glycaemic load and risk of the metabolic syndrome in a Korean population Br J Nutr 2008 100 576 584 10.1017/S0007114508904372 18328117

[B41] Volek JS Feinman RD Carbohydrate restriction improves the features of Metabolic Syndrome. Metabolic Syndrome may be defined by the response to carbohydrate restriction Nutr Metab 2005 2 31 10.1186/1743-7075-2-31 PMC1323303 16288655

[B42] Accurso A Bernstein RK Dahlqvist A Draznin B Feinman RD Fine EJ Gleed A Jacobs DB Larson G Lustig RH Dietary carbohydrate restriction in type 2 diabetes mellitus and metabolic syndrome: time for a critical appraisal Nutr Metab 2008 5 9 10.1186/1743-7075-5-9 PMC2359752 18397522

[B43] Bao J Atkinson F Petocz P Willett WC Brand-Miller JC Prediction of postprandial glycemia and insulinemia in lean, young, healthy adults: glycemic load compared with carbohydrate content alone Am J Clin Nutr 2011 93 984 996 10.3945/ajcn.110.005033 21325437

[B44] Lichtman SW Pisarska K Berman ER Pestone M Dowling H Offenbacher E Weisel H Heshka S Matthews DE Heymsfield SB Discrepancy between self-reported and actual caloric intake and exercise in obese subjects N Engl J Med 1992 327 1893 1898 10.1056/NEJM199212313272701 1454084

[B45] Prentice AM Black AE Coward WA Davies HL Goldberg GR Murgatroyd PR Ashford J Sawyer M Whitehead RG High levels of energy expenditure in obese women Br Med J (Clin Res Ed) 1986 292 983 987 10.1136/bmj.292.6526.983 PMC1339917 3083978

[B46] Willett WC Howe GR Kushi LH Adjustment for total energy intake in epidemiologic studies Am J Clin Nutr 1997 65 1220S 1228S 9094926 10.1093/ajcn/65.4.1220S

[B47] Kipnis V Subar AF Midthune D Freedman LS Ballard-Barbash R Troiano RP Bingham S Schoeller DA Schatzkin A Carroll RJ Structure of dietary measurement error: results of the OPEN biomarker study Am J Epidemiol 2003 158 14 21 10.1093/aje/kwg091 12835281

[B48] Lau C Toft U Tetens I Richelsen B Jorgensen T Borch-Johnsen K Glumer C Association between dietary glycemic index, glycemic load, and body mass index in the Inter99 study: is underreporting a problem? Am J Clin Nutr 2006 84 641 645 16960180 10.1093/ajcn/84.3.641

